# Adherence to Mediterranean dietary quality index and risk of breast cancer in adult women: a case-control study

**DOI:** 10.1186/s12905-023-02247-w

**Published:** 2023-03-14

**Authors:** Farhang Djafari, Parivash Ghorbaninejad, Fatemeh Dehghani Firouzabadi, Fatemeh Sheikhhossein, Hossein Shahinfar, Maryam Safabakhsh, Hossein Imani, Sakineh Shab-Bidar

**Affiliations:** 1 Department of Community Nutrition, School of Nutritional Sciences and Dietetics, Tehran University of Medical Sciences (TUMS), Tehran, Iran; 2 Department of Clinical Nutrition, School of Nutritional Sciences and Dietetics, Tehran University of Medical Sciences (TUMS), Tehran, Iran; 3 Student Research Committee, Iran University of Medical Sciences, Tehran, Iran; 4 Department of Nutrition, School of Public Health, Iran University of Medical Sciences, Tehran, Iran

**Keywords:** Breast cancer, Mediterranean dietary quality index, Case-control, MedDQI, Risk

## Abstract

**Background:**

Breast cancer (BC) is the fifth most prevalent cause of cancer-related deaths in Iran. Given that the role of whole-diet on cancer risk is important, this study aimed to assess the association of MedDQI and breast cancer risk.

**Methods:**

This hospital-based case-control study was performed on 150 women with pathologically confirmed breast cancer within the period of less than 3 months. Controls were 150 apparently healthy that were matched by age. Dietary data was collected using a validated questionnaire. To examine participants’ adherence to MedDQI, the MedDQI was created according to foods and nutrients highlighted or minimized in the MedDQI construction.

**Results:**

After adjusting for possible confounders, participants in the highest quartile of the MedDQI score had 55% lower odds of breast cancer than women in the bottom quartile (OR: 0.45, 95% CI: 0.21, 0.94, P trend: 0.02). Stratified analysis by menopausal status showed such association in postmenopausal women (OR: 0.24, 95% CI: 0.07, 0.8, P trend: 0.055) after controlling for age and energy intake.

**Conclusion:**

The results showed an inverse association between adherence to the MedDQI and risk of breast cancer among Iranian women. More prospective studies are needed to confirm our results.

## Introduction

Breast cancer (BC), the second-high prevalent cancer, is spreading around the world, especially in developing countries. Based on GLOBOCAN statistics for 2018, about 11.6% of women were recognized with breast cancer that year, with 626,679 associated deaths [1]. In Iran, BC is the fifth most prevalent cause of cancer-related deaths, including 24.4% of all cancers with a standard age rate (ASR) of 23.1 per 100,000 [2]. Several risk factors counting family history of BC, reproductive factors, and environmental factors contribute to the development of BC [3, 4]. The management and prognosis of metastatic breast cancer involve several metabolic aspects, including metabolic syndrome and obesity, glucose metabolism, and microRNA modulation [5]. Among the environmental factors, diet as a modifiable factor can play an important role in the development of BC [6], however, studies in this term are limited that according to some recent studies, adherence to the Mediterranean diet has been related to reducing risk of diseases such as cancers, hypertension, cardiovascular diseases, obesity, and diabetes [7–11]. Particularly, numerous studies found a negative association between the consumption of Mediterranean diet and risk of breast cancer [12–14]. Further, in a case-control study, authors tested the association between non-adherence to Mediterranean diet with lifestyle habits in the incidence.

of breast cancer. They found a clear positive relationship and synergism between non-adherence to Mediterranean diet with current smoker, physical inactivity, and alcohol consumption [15]. Previous studies have mostly assessed the associations between Mediterranean dietary pattern and the risk of diseases that do not consider categorizing sources of fat, protein, or carbohydrate, separately [16]. In this regard, focusing on different sources of fat, protein, or carbohydrate, considering their effects on the quality of the diet, can be a better approach to show the influence of the diet on the risk of diseases.

The Mediterranean dietary quality index (MedDQI), a beneficial tool for assessing the quality of the diet, was developed by Gerber et al. [17]. This index evaluates diet quality with an emphasis on different sources of fat (saturated fatty acids and cholesterol versus olive oil) and two different sources of protein (meat and fish) with the contrary scores, both on the poor and good sides, respectively. Given the effective role of different food sources in the development or prevention of cancer [18], focusing on the MedDQI can give a better picture of the association of the diet and BC risk. To the best of our knowledge, no study has investigated the association of the MedDQI and the risk of breast cancer. We, therefore, aimed to examine the potential association between the MedDQI with the risk of breast cancer in Iranian women.

## Materials and methods

### Study design and participants

**Fig. 1:**
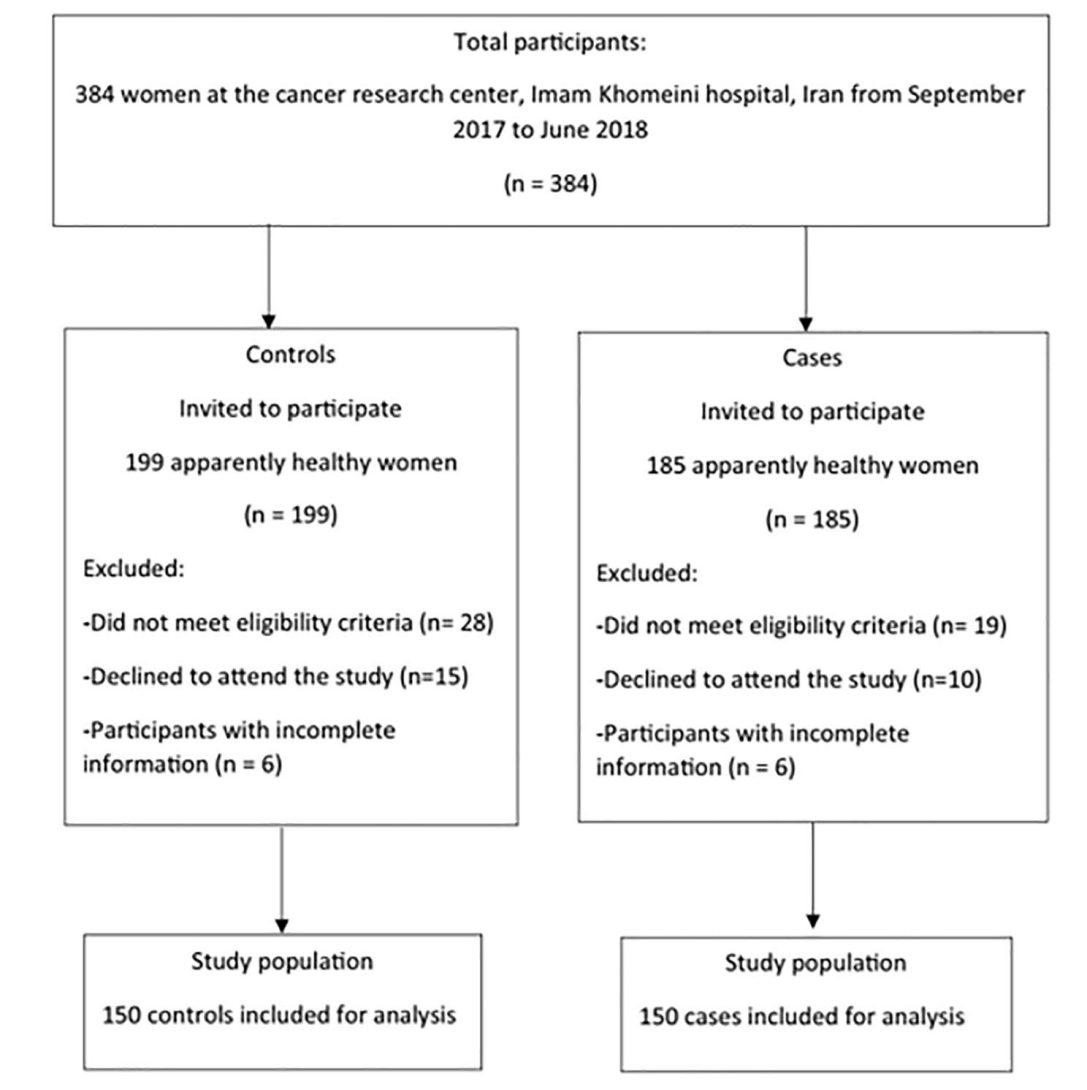
The flow diagram for participant selection

This hospital-based case-control study was conducted between September 23, 2017 and June 21, 2018 among Iranian women (46.6 ± 10.7 years old) who referred to cancer research center, Imam Khomeini Hospital in Tehran. To calculate the sample size calculation, the type I error of 5% and the study power of 95% was used. We hypothesized 5% of the difference in mean and SD of dietary grains between cases and controls and reached almost 150 patients with breast cancer and 150 healthy controls [19]. Cases (n = 150) who were suggested to participate in our study by a pathologist, were pathologically diagnosed with BC. While, controls (n = 150) were apparently healthy women among relatives of patients in other wards of cited Hospital, like dermatology, urology, orthopedic, etc., by poster installation. There was no relationship between these two groups and they were matched just by age. We included patients with a diagnosis period of lower than 3 months to minimize the effect of awareness of BC on patient’s dietary reports. Furthermore, subjects who had any other cancers’ history and long-term dietary restrictions and also controls with BC history were excluded (Fig. 1). The skilled interviewer recorded information on age (year), energy intake (kcal/d), education (university graduated, n (%)), urban-residency (yes, n (%)), family history of breast cancer (yes, n (%)), physical activity (Met/min/week), marital status (married, n (%)), smoking (never smoked, n (%)), alcohol consumption (never used, n (%)), dietary supplement use (yes, n (%)), length of breast-feeding (year), menopausal status (yes, n (%)), history of hormone replacement therapy (yes, n (%)) and BMI (kg/m^2^), throughout a 45-min structured face‐to‐face interview by a standard questionnaire.

### Dietary intake assessment

Usual dietary intake of women was evaluated using a valid and reliable 147-item Food Frequency Questionnaire (FFQ) [20] which included a list of groceries and a standard size of each food item. The trained dieticians asked the participants to recall their consumption frequency of each item on a daily, weekly, monthly, and annual basis. When the participants’ reports were not adaptive with the given portion sizes, they were asked to consider their own portion sizes. To estimate energy and nutrient intakes, the household measures and the USDA food composition database which modified for Iranian foods [21, 22], were used to convert the consumed food portion sizes to grams. The calculations were also performed by a modified version of NUTRITIONIST IV software for Iranian foods (version 7.0; N-Squared Computing, Salem, OR, USA).

### Construction of Med-DQI

We calculated the diet score based on the Mediterranean diet quality index (MedDQI) (Table 1). This dietary index includes 7 food components which were given a score of 0, 1 or 2 according to the daily intake of each item. Finally, a total score was obtained by summing up the scores of these food items and ranged from 0 to 14. To obtain lower score on this index shows a higher nutrition quality and following the Mediterranean dietary pattern [17]. To estimate the MedDQI score, we categorized participants based on quartile groups of the above-mentioned component’s intakes to minimize misclassification.

**Table 1:** Score formation of the Mediterranean Dietary Quality Index

Scoring	0	1	2
Saturated fatty acids (% energy)	< 10	10–13	> 13
Cholesterol (milligram)	< 300	300–400	> 400
Meats (gram)	< 25	25–125	> 125
Olive oil (milliliter)	> 15	15–5	< 5
Fish (gram)	> 60	60–30	< 30
Cereals (gram)	> 300	300–100	< 100
Vegetables + fruits (gram)	> 700	700–400	< 400

### Assessment of other variables

Weight was measured with light clothing and without shoes, by using a digital weighing scale (Seca725 GmbH & Co. Hamburg, Germany) to the nearest 100 g and the height was assessed while standing and keeping the shoulders and hips against the wall without shoes, using a stadiometer (Seca, Germany) with an accuracy of 0.1 cm. Body mass index (BMI) was calculated as weight divided by squared height and presented as kg/m^2^. A validated short from International Physical Activity Questionnaire [23] was used to assess subject’s physical activity levels. Recorded amounts were presented based on Metabolic Equivalents (METs)[23]. Then, the duration and frequency of physical activity days were multiplied by the MET value of the activity and sum of them was calculated as the total exercise minute per week.

### Statistical analyses

All individuals were categorized according to the quartiles of MedDQI score. We analysed the study participants’ characteristics and dietary intakes according to MedDQI score quartiles, using one-way analysis of variance (ANOVA) and χ^2^ tests for continuous and categorical variables, respectively. Data were shown as the mean ± SD for continuous variables and percent (%) for categorical ones. Odds ratio and 95% confidence intervals were obtained using logistic regression to determine the relationship of adherence to the MedDQI score with risk of breast cancer. The risk was reported in crude and 3 adjusted models including confounders such as age (year), energy intake (kcal/d), education (university graduated, n (%)), urban-residency (yes, n (%)), family history of breast cancer (yes, n (%)), physical activity (Met/min/week), marital status (married, n (%)), smoking (never smoked, n (%)), alcohol consumption (never used, n (%) ), dietary supplement use (yes, n (%)), length of breast-feeding (year), menopausal status (yes, n (%)), history of hormone replacement therapy (yes, n (%)) and BMI (kg/m^2^). In this analysis, the first quartile of exposure was considered as the reference category. All statistical analyses were done using the Statistical Package for the Social Sciences (SPSS version 22; SPSS Inc.). We considered p < 0.05 as the significance level.

## Results

The mean age of participants was 46.6 ± 10.7 year in both case and control groups. Moreover, the BMI of the participants in case and control groups were 28.1 ± 4.6 and 28.2 ± 5.2 kg/m^2^ respectively. General characteristics of the study subjects with and without BC are indicated in Table 2. Also, we showed this information for participants in this table. Women with BC were more likely to be older and they had a longer breastfeeding period than women without BC. Additionally, compared with the participants in the first quartile of the MedDQI, those in the top quartile had lower BMI. No significant differences were observed in other variables.

**Table 2:** Characteristics of the study subject across patients with and without breast cancer and also across the quartile categorize of the Mediterranean Dietary Quality Index (MedDQI).

	Breast cancer			Quartiles of Mediterranean Dietary Quality Index				
	Yes (n = 150)	No (n = 150)	P^*^	Q1 (n = 103) MedDQI score range: 3–5	Q2 (n = 91)	Q3 (n = 49)	Q4 (n = 57) MedDQI score range:8–11	P^*^
Age (y)	46.6 ± 10.7	46.6 ± 10.7	**< 0.001**	47.0 ± 10.5	47.1 ± 10.0	44.5 ± 10.5	46.6 ± 12.3	0.4
BMI (kg/m^2^)	28.1 ± 4.6	28.2 ± 5.2	0.8	28.70 ± 5.32	28.73 ± 4.66	27.84 ± 4.25	26.65 ± 4.97	**0.01**
Physical activity (Met/min/week)	475.8 ± 1043.2	590.0 ± 843.8	0.2	644.4 ± 970.2	406.8 ± 708	388.8 ± 561	655.8 ± 1389.6	0.8
Family history of breast cancer yes, (%)	30 (20)	39 (26)	0.2	21 (20.4)	20 (22)	12 (24.5)	16 (28)	0.8
University graduated n, (%)	21 (14)	28 (18.7)	0.6	19 (18.5)	13 (14.3)	8 (16.3)	9 (15.9)	0.3
Urban-resided n, (%)	140 (93.3)	139 (92.7)	0.8	99 (96.1)	85 (93.4)	46 (93.9)	49 (86)	0.1
Married, n (%)	129 (86)	136 (90.7)	0.2	86 (83.5)	83 (91.2)	46 (93.9)	50 (87.7)	0.5
Menopause status yes, n (%)	62 (41.3)	54 (36)	0.3	41 (39.8)	37 (40.7)	15 (30.6)	23 (40.4)	0.6
Age at first menarche (year)	14.4 ± 1.7	13.4 ± 1.6	0.2	14.7 (1.7)	13.6 (1.2)	13.2 (1.5)	13.6 (1.7)	0.2
Number of child, n	2.6 ± 2.1	2.4 ± 2.1	0.3	2.5 (1.9)	2.5 (2.4)	2.3 (1.6)	2.6 (2.3)	0.9
Length of breastfeeding (year)	4.2 ± 3.5	3.5 ± 2.7	**0.059**	4.07 (3.3)	3.8 (2.8)	3.3 (3.1)	4.2 (3.2)	0.8
History of HRT, n (%)	12 (8)	8 (5.3)	0.3	6 (5.8)	2 (2.2)	6 (12.2)	2 (10.5)	0.07
Smoking, never smoked, n (%)	147 (98)	144 (96)	0.3	101 (98.1)	87 (95.6)	47 (95.9)	56 (98.2)	0.7
Alcohol, never used, n (%)	149 (99)	148 (98)	0.5	102 (99)	90 (98.9)	48 (98)	57 (100)	0.5
Dietary supplement use, yes, n (%)	80 (53.3)	84 (56)	0.09	61 (59.2)	49 (53.8)	24 (49)	30 (52.6)	0.6
Medication use §,yes, n (%)	64 (42)	62 (41)	0.8	45 (43.7)	37 (40.7)	18 (36.7)	26 (45.6)	0.3
Comorbidities †, n (%)	38 (25)	31 (20)	0.3	46 (44.7)	42 (46.2)	26 (53.1)	29 (50.9)	0.3

BC, Breast Cancer

BMI, body mass index

HRT, hormone replacement therapy

kg/m2, kilogram/meter2

MET/min/wk, metabolic equivalent minute per week

^*^ANOVA for continuous variables and Chi-square test for categorical variables

§Lipid lowering and anti-hypertensive medications

†Diabetes, Hypertension and Hyperlipidemia

Dietary intakes of the patients across the case and control groups as well as across the quartiles of MedDQI are provided in Table 3. Compared to controls, women with BC consumed higher amounts of saturated fatty acids and total energy. In addition, participants in the highest quartile of MedDQI had higher intakes of saturated fatty acids, cholesterol, meat, and lower intakes of olive oils and total fruits and vegetables.

**Table 3:** Dietary intakes of study participants across case and control groups as well as across quartile categories of the Mediterranean Dietary Quality Index

	Breast cancer			Quartiles of Mediterranean Dietary Quality Index				
	Yes (n = 150)	No (n = 150)	P^*^	Q1 (n = 103) MedDQI score range: 3–5	Q2 (n = 91)	Q3 (n = 49)	Q4 (n = 57) MedDQI score range: 8–11	P^*^
Saturated fatty acids (% energy)	9.1 ± 4	8.1 ± 3	**0.02**	6.9 ± 1.9	7.7 ± 2.3	8.9 ± 2.7	12.7 ± 4.8	**< 0.001**
Cholesterol (mg/d)	255 ± 117	229 ± 126	0.06	194 ± 63	208 ± 74	269 ± 112	359 ± 179	**< 0.001**
Meats (gr/d)	51.9 ± 41.9	46.6 ± 31.5	0.26	37.3 ± 22.2	44.5 ± 30.4	57.3 ± 47.4	71.6 ± 64.2	**< 0.001**
Olive oil (ml/d)	1.9 ± 3.2	1.8 ± 3	0.82	3 ± 3.9	1.6 ± 2.7	0.8 ± 1.3	1 ± 2.4	**< 0.001**
Fish (gr/d)	8.9 ± 11.7	7.8 ± 9	0.39	9.4 ± 13.1	7.5 ± 8.4	6.6 ± 8	9.2 ± 9.7	0.63
Cereals (gr/d)	343 ± 288	495 ± 2390	0.44	633 ± 2875	299 ± 314	314 ± 225	314 ± 252	0.23
Vegetables + fruits (gr/d)	999 ± 413	974 ± 343	0.57	1,098,326	958 ± 331	929 ± 433	881 ± 447	**< 0.001**
Energy intake (kcal/d)	2914.1 ± 1159.0	2660.3 ± 799.6	**0.02**	3173.0 ± 1945.7	2888.0 ± 921.3	2980.4 ± 1040.9	2986.1 ± 995.7	0.06

Odds ratios and 95%CI of the BC across quartiles of the MedDQI are presented in Table 4. In the crude model, there was a significant inverse association between adherence to MedDQI and odds of BC (OR _fourth vs. first quartile_: 0.47, 95% CI: 0.23, 0.92, P trend: 0.01). After controlling for confounders, participants in the top quartile of Med-DQI score had 55% less likely to have BC compared with those in the bottom quartile (OR _fourth vs. first quartile_: 0.45, 95% CI: 0.21, 0.94, P trend: 0.022). These findings remained significant even after adjustment for BMI (OR _fourth vs. first quartile_: 0.45, 95% CI: 0.21, 0.94, P trend: 0.02).

**Table 4:** Risk for breast cancer according to quartiles of the Mediterranean Dietary Quality Index with stratification by menopausal status

	OR (95% CI)				
	Q1 MedDQI score range: 3–5	Q2	Q3	Q4 MedDQI score range: 8–11	P _for trend_
Total					
No. of cases/controls	52/51	37/54	22/27	39/18	
Crude	1	1.48 (0.84–2.63)	1.25 (0.63–2.47)	0.47 (0.23–0.92)	**0.012**
Model 1	1	1.49 (0.84–2.65)	1.25 (0.63–2.5)	0.47 (0.24–0.93)	**0.012**
Model 2	1	1.45 (0.79–2.65)	1.19 (0.58–2.45)	0.45 (0.21–0.94)	**0.022**
Model 3	1	1.45 (0.79–2.65)	1.45 (0.79–2.65)	0.45 (0.21–0.94)	**0.02**
Premenopause					
No. of cases/controls	32/30	21/33	14/20	21/13	
Crude	1	1.67 (0.8–3.51)	1.52 (0.65–3.54)	0.66 (0.28–1.54)	0.15
Model 1	1	1.69 (0.8–3.56)	1.54 (0.66–3.62)	0.67 (0.28–1.59)	0.16
Model 2	1	2.07 (0.94–4.58)	1.57 (0.63–3.91)	0.72 (0.28–1.8)	0.11
Model 3	1	2.06 (0.93–4.55)	1.57 (0.63–3.93)	0.73 (0.28–1.54)	0.12
Postmenopause					
No. of cases/controls	20/21	16/21	8/7	18/5	
Crude	1	1.69 (0.51–3.56)	0.83 (0.25–2.72)	0.26 (0.08–0.84)	0.07
Model 1	1	1.33 (0.53–3.34)	0.9 (0.27–2.99)	0.24 (0.07–0.8)	**0.055**
Model 2	1	1.22 (0.44–3.39)	1.05 (0.27–4.04)	0.23 (0.05–0.91)	0.1
Model 3	1	1.23 (0.44–3.46)	0.9 (0.25–3.88)	0.18 (0.04–0.77)	0.06

Model 1: Adjusted for age and energy intake

Model 2: Further adjusted for education, residency, family history of breast cancer, physical activity, marital status, smoking, alcohol consumption, supplement use, length of breast-feeding, menopausal status and history of hormone replacement therapy

Model 3: Further adjusted for BMI.

Stratified analysis by menopausal status expressed that after adjusting for age and energy intake, postmenopausal women with the highest adherence to the MedDQI had 76% lower odds for having BC than those with the lowest adherence (OR _fourth vs. first quartile_: 0.24, 95% CI: 0.07, 0.8, P trend: 0.055). There was no significant association between MedDQI and odds of BC in premenopausal women in crude or adjusted models.

(Model 1: Adjusted for age and energy intake; Model 2: Further adjusted for education, residency, family history of breast cancer, physical activity, marital status, smoking, alcohol consumption, supplement use, length of breastfeeding, menopausal status, and history of hormone replacement therapy; Model 3: Further adjusted for BMI)

## Discussion

In this hospital-based case-control study, we found a significant inverse association between MedDQI scores and odds of BC among Iranian women. This inverse association was also seen in postmenopausal women after controlling for energy intake and age. No significant relation was found between MedDQI scores and BC in premenopausal women. This is the first study looking at the link between the MedDQI scores and the risk of BC in Iranian women. BC is the most common malignancy in women [1] Statistics demonstrate a significant increase in the incidence of BC over the past 25 years worldwide [24]. Diet plays a considerable role in the primary prevention of BC. In the present study, adherence to a diet with low MedDQI scores was inversely associated with odds of BC. The effects of a non-Mediterranean diet in the incidence of breast cancer is also well established [25]. Consumption of fresh fruits and vegetables increase the consumer polyphenols that help in fighting against tumorigenesis. In the other hand increased intake of fiber and carbohydrate may be associated with the breast cancer prognosis. Soybean proteins consumption are involved in breast cancer risk reduction and lowering the chances of breast cancer reoccurrence. Ethanol consumption exert their carcinogenic effect on breast tissues and mediate breast cancer development. Processed meat releases the carcinogens compound likes heterocyclic amines, which mediate the onset of breast cancer. High saturated fat diet increases receptor-positive cancer, particularly ER + risk of breast cancer [25]. This relationship was found in postmenopausal women after taking potential cofounders into account. Studies looking at the link between healthy eating habits and the BC have produced conflicting results [26–29]. Similar to our research, several studies have found an inverse relationship between the risk of BC and good eating habits, or diets with low MedDQI scores [26]. Similar to our study, a recent meta-analysis revealed that following a Mediterranean-style diet may help lower the risk of breast cancer, albeit this link was not significant in premenopausal women [30]. Moreover, in an updated meta-analysis conducted by Morze et al. the highest adherence to MedDiet was inversely associated with cancer mortality and BC risk [31]. An increased adherence to the prudent or similar dietary patterns, those rich in fruit, vegetables, fish, whole grains, and low-fat dairy products, were strongly associated with a reduced risk of BC, according to a recent meta-analysis of 32 observational studies looking at the associations between various dietary patterns and odds of BC. These connections were similarly significant in premenopausal women, despite our study, though. Additionally, a Turati study revealed that following a Mediterranean diet was linked to a lower incidence of BC in both pre- and post-menopausal women [12]. The small sample size (n = 300) in this subgroup could be the reason why no correlation between MedDQI scores and risk of BC in premenopausal women was found. We did not look at the associations between BC subtypes. The relationship between following a diet with low Med-DQI scores and lowered risk of a specific subtype of BC has been discussed in several papers [12, 32, 33] In a prospective cohort study, a Mediterranean diet was associated with decreased risk of estrogen and progesterone receptor-negative (PR- ER-) BC [12]. According to another cohort study, following a Mediterranean diet reduced women’s likelihood of developing (ER-)-type BC [32]. A cohort study that involved 49,258 generally healthy women showed that adopting the Mediterranean diet did not significantly lower the risk of BC, in contrast to the current study [28]. Some research in this area has not discovered any conclusive links between healthy eating habits or other similar dietary patterns and the risk of BC [34–36]. The lack of several confounders being controlled for in some research may account for this disparity. Additionally, various dietary evaluation methods and components used in different research may contribute to the contradictory results. Based on the case-control nature of this study, the patients might have reported their current dietary intakes rather than their usual diet. This would result in a biased association between the MedDQI scores and risk of BC compared to those reported in prospective cohort studies, in which the exposure has been measured prior to disease incidence. Second, we ascertained the MedDQI scores based on dietary intakes of both cases and controls, while cases might had changed their dietary intakes after disease manifestation. This would lead to a higher adherence to MedDQI scores in cases than controls and would eventually result in a biased relationship. Finally, no information about subtypes of BC is collected in this study. It has been indicated that diet with low MedDQI scores may have an impact on some subtypes of BC and no effect on others. The underlying mechanisms for the possible favorable of the diet with high MedDQI scores and odds of BC are not completely known. The MedDQI scores is a valuable tool to predict dietary quality and has been already verified using nutritional biomarkers [17]. This index was founded on the recommendations by the National Research Council (NRC) and American Heart Association (AHA) about the diet and health [37]. The intake of 30% or less of the daily total energy from fat, 10% or less of the total energy derived from saturated fat, 30 mg/d or less from cholesterol, 55% of energy from complex carbohydrates and 5 servings or more from fruits and vegetables have been taken into account by NRC and AHA. Fruit, vegetables and whole grains are good sources of antioxidants, dietary fiber and polyphenols, which can mediate the inverse association between the Dietary Approaches to Stop Hypertension (DASH) diet in relation to BC [38]. The consumption of olive oil, due to its monounsaturated fatty acids, have shown to be beneficial associated with some types of BC prevention and survival [39]. In spite of the fact that this study has demonstrated that dietary habits and lifestyle have an impact on the incidence of breast cancer, there is no doubt that hormones are the main cause of the disease. Efforts must also be made to encourage a healthy lifestyle, with special emphasis on the importance of eating a diet consisting primarily of fruits, vegetables, and whole grains with a low intake of red meat and saturated fats. To build the foundation for future advances in evidence-based public health efforts in this region, continued and expanded research on diet, lifestyle and breast cancer risk is urgently necessary.

Suitable sample size, careful assessment of confounding elements and their controlling in the analyses and being the first investigation in Middle East women could be considered as the strengths of the present study. However, some limitations should be considered. First, the case control design of the study does not permit us inferring causality. In addition, these kinds of studies are subject to selection and recall bias. In case-control studies, cases may report their past diet correctly due to their cancer diagnosis. This can attenuate this association. Another concern for case-control studies is that cases may have changed their diet before diagnosis due to early symptoms of the disease. To reduce this error, we recruited newly-diagnosed cases in the study. Furthermore, we used FFQ for assessing dietary intakes which can result in misclassification in our study participants. In addition, an energy adjusted MedDQI scores was applied which could reduce the possibility of subject misclassification. However, we used a qualified questionnaire to evaluate dietary intakes. Finally, we did not collect information about estrogen or progesterone receptor status of study patients.

## Conclusion

In conclusion, our findings showed an inverse association between diet with high MedDQI scores and odds of BC among Iranian women. More cohort studies are needed to approve our findings.

## Data Availability

The datasets generated or analyzed during the current study are not publicly available but are available from the corresponding author on reasonable request.
